# A Comparative Study of Encapsulation of β-Carotene via Spray-Drying and Freeze-Drying Techniques Using Pullulan and Whey Protein Isolate as Wall Material

**DOI:** 10.3390/foods13121933

**Published:** 2024-06-19

**Authors:** Christina Drosou, Magdalini Krokida

**Affiliations:** School of Chemical Engineering, National Technical University of Athens, Zografou Campus, 9 Heroon Polytechniou St., 15780 Athens, Greece; mkrok@chemeng.ntua.gr

**Keywords:** atomization, β-carotene stability, carotenoids, degradation kinetics, lyophilization

## Abstract

The encapsulation of β-carotene was investigated using pullulan and whey protein isolate (WPI) as a composite matrix at a weight ratio of 20:80, employing both spray-drying and freeze-drying techniques. The influence of processing parameters such as the concentration of wall material, flow rate, and inlet temperature for SP encapsulants, as well as wall-material concentration for FZ encapsulants, was examined in terms of encapsulation efficiency (EE). The morphology, structural characterization, moisture sorption isotherms, and thermal properties of the resulting encapsulants at optimum conditions were determined. Their stability was investigated under various levels of water activity, temperature conditions, and exposure to UV–Vis irradiation. β-carotene was efficiently encapsulated within SP and FZ structures, resulting in EE of approximately 85% and 70%, respectively. The degradation kinetics of β-carotene in both structures followed a first-order reaction model, with the highest rate constants (0.0128 day^−1^ for SP and 0.165 day^−1^ for FZ) occurring at an intermediate water-activity level (*a*_w_ = 0.53) across all storage temperatures. The photostability tests showed that SP encapsulants extended β-carotene’s half-life to 336.02 h, compared with 102.44 h for FZ encapsulants, under UV–Vis irradiation. These findings highlight the potential of SP encapsulants for applications in functional foods, pharmaceuticals, and carotenoid supplements.

## 1. Introduction

Recent research has shown significant interest in extracting food ingredients such as carotenoids, gingerols, carminic acid, capsaicin, and sweeteners from natural sources [1,2]. Among these, carotenoids are natural pigments that are derived from various sources, including fruits, vegetables, and microorganisms. The most commonly used carotenoids in various applications include β-carotene, lutein, zeaxanthin, astaxanthin, and lycopene. These carotenoids are widely employed in the food, animal feed, pharmaceutical, and cosmetics industries, among others, for their various applications and functionalities [3]. The most common properties of carotenoids include their vibrant colors, antioxidant effects, provitamin A activity, and lipophilic nature. Among carotenoids, β-carotene is the most widely used in different fields due to its cyclic structure containing 11 conjugated double bonds, contributing to its high antioxidant activity. β-carotene, a prominent carotenoid, has seen substantial market growth due to its widespread applications in various industries. The global β-carotene market was valued at approximately USD 500 million in 2020, with a projected compound annual growth rate (CAGR) of around 4% from 2021 to 2026. This growth has been driven by increasing consumer awareness of the health benefits associated with β-carotene, such as its antioxidant properties and role as a precursor to vitamin A. Major production methods include chemical synthesis, extraction from natural sources, and microbial fermentation, with advancements in biotechnological processes improving yield and purity [4]. In the food industry, β-carotene is extensively used as a natural colorant and nutrient fortifier in products like margarine, beverages, and dietary supplements [5]. The pharmaceutical sector incorporates β-carotene into supplements to enhance immune health and reduce the risk of chronic diseases [6]. Additionally, the cosmetics industry utilizes β-carotene in skincare products for its anti-aging and skin-protective effects [7]. The animal feed industry also benefits from β-carotene, as it enhances the pigmentation of egg yolks, poultry skin, and fish flesh, making products more appealing to consumers [8]. These diverse applications underscore the importance of β-carotene across multiple sectors, driving ongoing research and development to further optimize its production and use.

Given its extensive use and market growth, especially in the food industry, enhancing the stability and bioavailability of β-carotene in food products is of paramount importance. β-carotene, owing to its unsaturated chemical structure, is susceptible to oxidative degradation and isomerization when exposed to heat, light, and oxygen. This susceptibility can result in reduced bioavailability and physiological activity, as well as diminished color in food products. Additionally, β-carotene is insoluble in water and it cannot be directly incorporated into aqueous-based formulations [9]. Therefore, efforts have been directed towards enhancing the utilization, bioavailability, and shelf life of β-carotene in food products, with encapsulation emerging as a vital technique to improve its physical and chemical stability during processing and storage.

Encapsulation serves as an efficient approach for enhancing the stability of β-carotene via enveloping the core material within a protective coating, often referred to as a wall material. Notably, various encapsulation techniques have been proposed to make β-carotene feasible in the food industry. Among these techniques, spray drying and freeze drying stand out as the most frequently employed methods, resulting in the development of dry encapsulated structures in powder form [10]. Freeze drying, in particular, is highly effective for encapsulating thermosensitive substances, as it minimizes thermal degradation reactions. Despite its high operational expenses due to its substantial energy demands and extended processing duration, freeze drying is commonly utilized for encapsulating carotenoids [11]. On the other hand, spray drying stands as the predominant method employed in the food industry due to its flexibility, continuous operation, and cost-effectiveness. The encapsulation mechanism involves surrounding β-carotene with a protective wall material through spray-drying and freeze-drying techniques. In spray drying, the β-carotene emulsion is atomized into a hot drying chamber, where the solvent rapidly evaporates, forming dry particles encapsulating β-carotene. Freeze drying, on the other hand, involves freezing the β-carotene emulsion and then reducing the surrounding pressure to allow the frozen solvent to sublimate directly from the solid to the gas phase, forming a porous structure that encapsulates the β-carotene. Both techniques aim to protect β-carotene from oxidative degradation and enhance its stability.

Recent advancements in the encapsulation of β-carotene using spray-drying and freeze-drying techniques have highlighted significant improvements in stability, bioavailability, and application efficiency. Tucumã oil containing high amounts of carotenoids was encapsulated in gum arabic through SP, resulting in increased oxidative stability under high temperature and high carotenoid retention after storage [12]. In another study, the application of octenyl succinic anhydride (OSA) starch and chitosan to form multilayer nanoemulsions in the spray drying microencapsulation of β-carotene in oil was examined. These multilayer emulsions provided better stability and protection during storage [13]. Additionally, the powders obtained from spray drying highly hydrophilic solids emulsions with a trehalose-high DE maltodextrin mixture when used as glass formers (both single-layer and layer-by-layer interfaces) effectively protected encapsulated lutein and all-trans-β-carotene during storage at different temperatures (35 °C, 50 °C, and 65 °C) [14]. A spray-dried microencapsulation process significantly improved the stability of lutein-rich extract from *Chlorella vulgaris*. The powder retained approximately 65% of lutein, twice the retention of the raw extract [15]. Furthermore, microencapsulation of carrot waste in WPI and inulin was performed and the encapsulation efficiencies were 63.69% for FZ and 53.78% for SP. The FZ encapsulate exhibited better stability in terms of hygroscopicity, oxidative stability, and color properties compared with the SP encapsulate [16]. In another study, the potential of amaranth grain starch and protein-rich fractions as wall materials was evaluated for use in microcapsules designed to carry β-carotene. The results indicated that protein-based microcapsules demonstrated better β-carotene storage stability compared with starch-based microcapsules, especially at temperatures of 8 °C and 25 °C [17].

The choice of wall material is a crucial factor in the encapsulation process. Notably, the selection of an appropriate wall material significantly impacts both the efficiency of encapsulation and the properties of the resulting powder [18]. Typically, carotenoids are encapsulated using either synthetic or natural polymers, with polysaccharides and proteins being the most frequently used in spray-drying and freeze-drying encapsulation for food formulations. The use of gum arabic [12], maltodextrins [19], modified starches [20], proteins [21] and their combinations [11,15,16] as wall materials has been extensively studied for the encapsulation of carotenoids, employing both techniques. Pullulan, a water-soluble polysaccharide comprising repeating maltotriose units linked via α-1,6-glycosidic bonds, exhibits potential as a wall material for carotenoid encapsulation due to its ability to form oxygen-impermeable structures, crucial for mitigating β-carotene degradation. However, pullulan exhibited poor emulsifying capacity leading to decreased encapsulation efficiency and stability when employed as a wall material for applications involving lipophilic compounds [9]. WPI, a commercial whey protein product, is a mixture of globular proteins, with β-lactoglobulin and α-lactalbumin being the main components. WPI is widely used as an emulsifier in the food industry and it has demonstrated efficacy as a carrier, either independently or in conjunction with other polymers, for encapsulating carotenoids, yielding structures characterized by improved stability [22]. Pullulan and WPI were selected as wall materials due to their complementary properties. This combination aimed to leverage the barrier properties of pullulan and the emulsifying efficiency of WPI to enhance encapsulation efficiency and the stability of β-carotene. An important consideration in the spray-drying process is the potential generation of undesired compounds such as melanoidins, particularly when WPI is combined with sugars at high temperatures. Melanoidins are formed through Maillard reactions and can affect the sensory properties and bioactivity of the final powder [23]. The formation of melanoidins not only impacts the color and flavor of the encapsulated product but can also alter its nutritional and functional properties. Therefore, careful selection of wall materials and optimization of processing conditions are crucial to minimize the formation of these compounds and preserve the bioactivity of the encapsulated carotenoids. Another critical aspect is the need for the use of optimal ratios to prevent phase separation and ensure efficient encapsulation of β- carotene. In the literature, the use of pullulan and WPI as composite wall materials has been investigated for encapsulating astaxanthin through freeze drying and β-carotene via coaxial electrospinning and electrospraying processes, presenting a promising avenue for enhancing the stability of carotenoids [9,24,25]. To the best of our knowledge, there have been no previously published reports comparing the effectiveness of pullulan and WPI as matrices for producing β-carotene-loaded spray-dried (SP) and freeze-dried (FZ) encapsulants nor their ability to retain β-carotene stability during storage.

The aim of this study was to encapsulate β-carotene using a composite wall material consisting of pullulan and WPI at a weight ratio of 20:80, through both spray-drying and freeze-drying techniques. It also aimed to compare the two encapsulation techniques, SP and FZ, in terms of their encapsulation efficiency, morphology, moisture content, thermal properties (glass transition temperature), and the β-carotene stability of the resulting encapsulants. Furthermore, the degradation kinetics of SP and FZ encapsulants were investigated under various water-activity levels, temperature conditions, and exposure to UV–Vis irradiation.

## 2. Materials and Methods

### 2.1. Materials

β-Carotene (synthetic, ≥93% (UV), powder) was supplied by Merck SA (Athens, Greece). WPI, purity 97.0%–98.4%, and pullulan (IP 20), purity > 90%, were provided by JMN Pharmaceutical (Athens, Greece) and Hayashibara Biochemical Lab. Inc. (Okayama, Japan), respectively. All other chemicals were of analytical grade and purchased from Merck SA (Athens, Greece).

### 2.2. Preparation of β-Carotene Emulsions for Spray Drying and Freeze Drying

Aqueous solutions of pullulan and WPI (pH = 3.0) were prepared at a weight proportion of 80:20 *w*/*w* via mixing with a magnetic stirrer (RSM-14HP, Phoenix Instrument GmbH, Garbsen, Germany) for at least 4 h and 500 min^−1^ at room temperature (25 °C) to ensure complete solubilization of the polymers. An oil-in-water emulsion was then prepared through mixing 1.5% *w*/*w* aqueous solution of WPI (pH = 3) and β-carotene corn oil suspension (0.25 mg/mL) using a high-speed homogenizer (T 25 digital ULTRA-TURRAX, IKA, Staufen, Germany) equipped with an S25N-18G dispersing tool, adjusting the electronic speed control to 10,500 rpm for 5 min. The resulting emulsion was passed through an ultrasonic system (Nanjing Xianou Instruments Manufacture Co., Ltd., Nanjing, China) operating at 720 W for 5 min in order to reduce the droplet size of the emulsion and the degree of dispersion. The final emulsions, which were used as feed solutions in the spray drying and freeze drying, were prepared through mixing the aqueous solution of the biopolymers (pullulan and WPI) and the emulsion (15% *w*/*w* in β-carotene oil suspension) in a magnetic stirrer for about 30 min. In the final emulsions, the concentration of the dispersed oil phase was adjusted to 5% *w*/*w*.

### 2.3. Characterization of the β-Carotene Emulsions

The viscosity of the β-carotene emulsions was measured according to the method described in our previous study [26]. The stability of the β-carotene emulsions was determined via the phase separation method. Specifically, 25 mL of the prepared emulsion was transferred to calibrated glass tubes and stored for 24 h at room temperature. After separating the emulsions into distinct phases, the height of the top layer (*H*_1_) was measured, and the emulsion stability index (*ESI*) was calculated using Equation (1):

(1) $$ESI\;\left(\%\right)=100\times \frac{H_{0}-H_{1}}{H_{0}},$$

where *H*_0_ is the initial height of the emulsion.

The size distribution of the β-carotene emulsion droplets was analyzed using a S3500 size distribution analyzer (Microtrac MRB, York, PE, USA) and quantified via the mean diameter of the emulsion droplets (*d*_43_), a robust indicator due to its sensitivity to small alterations in droplet size distribution. The size distribution analyzer operated based on light scattering (780 nm), with a recording range of average diameter values from 0.02 to 2800 μm. Measurements were conducted at ambient temperature, and the resulting data were corrected for refractive indices and the dielectric constant of the samples. The refractive indices used for water and corn oil were 1.330 and 1.461, respectively. The mean diameter of the emulsion droplets (*d*_43_) was expressed as a function of the droplet diameter, using Equation (2):

(2) $$d_{43}=\frac{\sum_{i}n_{i}d_{i}^{4}}{\sum_{i}n_{i}d_{i}^{3}},$$

where *d*_i_ is the diameter of the droplet.

### 2.4. Encapsulation of β-Carotene

#### 2.4.1. Spray Drying

The development of spray-dried encapsulated β-carotene structures (SP encapsulants) was stimulated via introducing the prepared emulsions in a spray dryer (Model YC-015, Shanghai Pilotech Instrument & Equipment Co., Ltd., Shanghai, China) operating with parallel flow, following the procedure described in our previous study [27]. Emulsion concentration, emulsion flow rate, and air inlet temperature were set as independent factors within the ranges of 4–8% *w*/*w*, 200–800 mL/h, and 160–190 °C, respectively, for use in the optimization process using response surface methodology (RSM) with central composite design (CCD). The resulting experimental design is presented in Table 1. The encapsulated powder was collected and stored in amber-colored glass bottles at −30 °C until further analysis.

#### 2.4.2. Freeze Drying

The concentration of the emulsion wall material was studied for the creation of freeze-dried β-carotene encapsulation structures (FZ encapsulants). Specifically, the pullulan/WPI polymeric blend concentration ranged from 4 to 8% *w*/*w* at a constant weight proportion of 20:80 *w*/*w* for the two materials. The emulsions were frozen for 24 h at −30 °C and then placed in a freeze dryer (Leybold-Heraeus GT 2A, Koln, Germany), where they remained for 48 h in plastic containers at a pressure of *p* = 0.2 mbar. Finally, the lyophilized samples were triturated in a mortar and kept at −30 °C until further examination.

### 2.5. Characterization of Encapsulants

#### 2.5.1. Encapsulation Efficiency

The encapsulation efficiency (EE, %) of SP and FZ encapsulants was determined via quantifying the β-carotene content entrapped within the structures and then dividing this value by the initial β-carotene content in the emulsion before the drying process. The determination of β-carotene content in SP and FZ encapsulants was performed using UV–Vis spectrophotometry measurements after extracting β-carotene from the SP and FZ structures. Free β-carotene was initially removed from the surface of the encapsulated structures using hexane solvent extraction. Approximately 0.1 g and 0.5 g of SP and FZ encapsulants, respectively, were mixed with 5 mL of hexane in a test tube, vortexed for 2 min, and then centrifuged at 3000 rpm for 3 min to remove the supernatant enriched with surface β-carotene. This process was repeated until the supernatant became colorless. Subsequently, the washed structures were disrupted in 3 mL distilled water until complete solubilization of structures in order to release their β-carotene content. Then, 5 mL ethanol and 2 mL hexane were added to the test tube and vortexed for 2 min. After centrifugation at 3000 rpm for 3 min, the supernatant (containing β-carotene) was collected and stored for analysis. The precipitate was resuspended in 1 mL ethanol and 2 mL hexane, following the same procedure until no β-carotene signal was obtained. Finally, the β-carotene concentration in the samples was determined spectrophotometrically at 458 nm using a UV-M51 spectrophotometer (BEL Engineering, Monza, Italy). The difference in the mass of material used was due to the difference in loaded β-carotene in the structures, ensuring that measurements remained within the limits of detection when analyzed spectrophotometrically.

#### 2.5.2. Yield of Encapsulation Process (Y)

To assess the performance of the encapsulation processes, the yield (Y, %) was determined. Y was determined via measuring the dry weight of the encapsulated solid mass and dividing it by the total amount of solid mass present before encapsulation. This ratio was then expressed as a percentage to indicate the Y of the encapsulation process.

#### 2.5.3. Scanning Electron Microscopy (SEM)

The microstructure of the SP and FZ samples was determined using scanning electron microscopy (SEM). Prior to SEM, the samples underwent gold plating using a SC7620 Mini Sputter Coater (Quorum Technologies, West Sussex, UK) for 90 s. The samples were then imaged at magnifications of 2500× for SP and 50× for FZ using a Quanta 200 (FEI, Hillsboro, OR, USA) Scanning Electron Microscope, using a large field detector (LFD) at an accelerating voltage of 12.5 kV.

#### 2.5.4. Attenuated Total Reflectance Infrared Spectroscopy (ATR-FTIR)

ATR-FTIR spectroscopy was employed to analyze the presence of materials and the chemical characteristics of SP and FZ encapsulants. The β-carotene oil suspension, the dry form of pullulan/WPI (20:80), and the SP and FZ encapsulants were scanned at operating wavelengths in the range between 4000 and 700 cm^−1^ using a Fourier-transform infrared spectrophotometer (FT/IR-4200, JASCO International Co., Ltd., Tokyo, Japan) with an attenuated total reflection (ATR) unit attached (ATR PRO-410-S, JASCO International Co., Ltd., Tokyo, Japan). A resolution of 4 cm^−1^ was used and each spectrum was acquired from 52 scans.

#### 2.5.5. Moisture Sorption Isotherms

In order to determine the moisture sorption isotherms of SP and FZ encapsulants, ~0.5 g dried samples were loaded in glass vessels and stored in glass desiccators at varying relative humidity levels, ranging from 0.11 to 0.95, across three temperatures (25 °C, 35 °C, 45 °C), in darkness. The desired relative humidity levels were achieved using oversaturated salt solutions, following the method outlined by Drosou et al. (2022) [9]. To inhibit microbial growth at high levels of water activity levels (aw > 0.53), a small amount of phenol was added to the desiccators. The samples were weighed at regular intervals until equilibrium was attained. The time required to reach equilibrium was approximately three weeks for all samples. Moisture content of the samples was determined after drying the samples at 130 °C for 1 h; moisture content was calculated as the amount of water divided by the dry weight of sample and expressed as g H_2_O/100 g dry solid. The amount of water was determined by subtracting the dry weight from the initial weight. Triplicate determinations were performed for the equilibrium moisture contents of each sample at every temperature.

The *a*_w_ moisture data were used to construct sorption isotherms. The data were fitted to the Brunauer–Emmett–Teller (BET) [28] or the Guggenheim–Anderson–DeBoer (GAB) [29] sorption isotherm models. The BET and GAB models are described in our previous study [9]. Nonlinear regression was used to determine the models’ constants via StatSoft STATISTICA 12.0 software.

#### 2.5.6. Differential Scanning Calorimetry (DSC)

The glass transition temperatures (*T*_g_) of the SP and FZ encapsulants were determined through DSC analysis (Pyris DSC-6, Perkin Elmer Ltd., Shelton, CT, USA) using Pyris 6 software version 4.01. Dried samples of approximately 20–30 mg were loaded into aluminum pans and kept in desiccators of different water-activity levels at 25 °C, 35 °C, and 45 °C to be re-humidified, until equilibrium was attained (approximately two weeks). Then, the aluminum pans were sealed hermetically and inserted into the calorimeter. An empty hermetically sealed pan was used as reference. Nitrogen gas with a constant flow rate of 20 L/min was used to create an inert atmosphere. The sample and reference pan were heated at a constant rate of 5 °C/min. The scanning conditions included the retention of samples for 2 min at 0 °C, heating from 0 °C to 10–15 °C above the onset temperature of the endothermic transition at a constant rate of 5 °C/min, instant cooling with liquid nitrogen, and heating from 0 °C to 200 °C at a constant rate of 5 °C/min. The glass transition temperature was determined during the second heating scan. All measurements were performed in three replications.

### 2.6. Storage Stability

In order to study the kinetic degradation of β-carotene during storage at various levels of water activity and temperature conditions, the SP and FZ encapsulants were placed into chambers with various relative humidity levels (*a*_w_: 0,11, 0,33, 0, 53, 0.75, 0.95) at three temperatures (25 °C, 35 °C, 45 °C) for 4 months. At fixed time intervals (5, 10, 15, 20, 30, 45, 60, 90, 120 days), β-carotene content was measured using a UV-M51 spectrophotometer (BEL Engineering, Monza, Italy) at a wavelength of 458 nm, as described in Section 2.5.1. The determination of the degradation constant rate (*k*) and half-life period (*t*_1/2_) of β-carotene was conducted using a first-order reaction kinetic model, as described in Equation (3):

(3) $$C=C_{o}e^{-kt},$$

where *C*_o_ is the initial concentration of β-carotene in SP and FZ encapsulants immediately after encapsulation process.

The dependence of reaction rate constants on temperature was modeled using the Arrhenius equation (Equation (4)). According to the Arrhenius formalism, a linear relationship exists between ln*k* and 1/*T*:

(4) $$k=k_{o}exp(-E_{a}/RT),$$

where *R* is the gas constant and *E*_a_ is the activation energy.

### 2.7. Photostability by UV–Vis Irradiation

SP and FZ encapsulants were placed into a chamber with a power density of 1000 W × m^−2^ (8 OSRAM-Ultravitalux lamps, 300 W) for 400 min to accelerate the oxidation of β-carotene. At fixed time intervals (15, 30, 60, 75, 90, 120, 180, 240, 300, 400 min), β-carotene content was measured using a UV-M51 spectrophotometer (BEL Engineering, Monza, Italy) at a wavelength of 458 nm, as described in Section 2.5.1. The photostability of SP and FZ encapsulants under UV–Vis irradiation was determined via calculating the photo-oxidation rate constant (*k*) and half-life periods (*t*_1/2_) of β-carotene.

### 2.8. Experimental Design and Statistical Analysis

RSM was employed to analyze the experimental data, resulting in the optimization and formulation of spray drying for encapsulating β-carotene. This was implemented using a commercial statistical package, StatSoft STATISTICA 12.0 (Hamburg, Germany). CCD of the RSM was used to develop the best possible levels of the independent variables. Three replications were performed for each independent experiment. The independent variables used were the wall-material concentration of the emulsion (% *w*/*w*, *Χ*_1_), the emulsion flow rate (mL/h, *X*_2_), and the air inlet temperature (°C, *X*_3_). These variables were coded at three levels, −1, 0, and 1. The dependent variable was β-carotene encapsulation efficiency (EE). Table 1 presents the responses for each combination of variables according to the full-design experimentation (16 runs). The generalized second-order quadratic equation proposed to express the response as a function of the independent variables is given as follows:

(5) $$Y=b_{0}+b_{1}X_{1}+b_{2}X_{2}+b_{3}X_{3}+b_{11}X_{1}^{2}+b_{22}X_{2}^{2}+b_{33}X_{3}^{2}+b_{12}X_{1}X_{2}+b_{13}X_{1}X_{3}+b_{23}X_{2}X_{3},$$

where Y is the dependent variable, *b*_0_ is a constant, *b*_1_, *b*_2_, and *b*_3_ are linear coefficients, *b*_11_, *b*_22_, and *b*_33_ are the quadratic coefficients, *b*_12_ the interaction coefficient of variables 1 and 2, *b*_13_ the interaction coefficient of variables 1 and 3, *b*_23_ the interaction coefficient of variables 2 and 3, and *X*_1_, *X*_2_, and *X*_3_ are independent variables.

Using analysis of variance (ANOVA), the data were assessed to determine the significant (*p* < 0.05) differences among all independent variables for encapsulated β-carotene. The variable with the lowest *p*-value indicated the most significant (*p* < 0.05) impact on the response. Additionally, after selecting the variables’ effect levels on responses, coefficients that were not significant (*p* > 0.05) were removed from the initial model, except when the quadratic and interaction influence along with that factor were significant. Consequently, the experimental data were analyzed using the reduced model eliminating non-significant values [30].

The significance test was conducted at a 95% limit, based on the total error criteria. Analysis of variance (ANOVA) was employed to determine the significance level for each response variable. The lack of fit test evaluated the agreement of mathematical models, with the F-value indicating its significance in model fitting. The efficiency of the model was evaluated using the R^2^ value [31]. The surface plot provided an optimal visual representation of the influence of independent variables, utilizing three-dimensional visualization to confirm the interaction between factors.

Furthermore, one-way analysis of variance (ANOVA) was applied in order to analyze the differences among the samples. Tukey’s test (α = 0.05) was applied and all the statistical tests were performed using StatSoft STATISTICA 12.0 software (Hamburg, Germany).

## 3. Results & Discussion

### 3.1. Optimization of Encapsulation Efficiency (EE, %) of SP Encapsulants Using RSM

The compatibility of pullulan and WPI as wall materials plays a crucial role in the formation and stability of β-carotene encapsulates. Pullulan, a polysaccharide, is known for its excellent film-forming properties and oxygen barrier capabilities, which help in protecting β-carotene from oxidative degradation. WPI, on the other hand, provides emulsifying properties that are essential for creating stable emulsions with β-carotene [9]. The interaction between pullulan and WPI can form a cohesive matrix that effectively traps and stabilizes β-carotene within the encapsulates.

From a chemical perspective, the hydrophilic nature of pullulan complements the amphiphilic properties of WPI, leading to improved dispersion of β-carotene in the aqueous phase and enhanced encapsulation efficiency [32]. Structurally, the combination of pullulan and WPI can result in a uniform and dense matrix that minimizes the exposure of β-carotene to environmental factors such as light, oxygen, and heat, thereby enhancing its stability [33]. Additionally, the compatibility of these materials ensures that the encapsulates have desirable physicochemical properties, such as appropriate moisture content and glass transition temperature, which are critical for maintaining the integrity of the encapsulated β-carotene during storage and handling.

In this study, RSM was employed to identify the optimal conditions for maximizing EE, taking into account the synergistic effects of pullulan and WPI. The effects of wall-material concentration (% *w*/*w*, *Χ*_1_), emulsion flow rate (mL/h, *X*_2_), and air inlet temperature (°C, *X*_3_) were determined for the development of SP encapsulants with high entrapment of β-carotene inside their structures through a spray-drying process. Table 1 presents the experimental data for the response variable (EE, %) for all individual trials in the experimental design. According to the results, the EE ranged from 62.00 ± 0.25% to 83.54 ± 0.57%, indicating that β-carotene can be effectively encapsulated in SP pullulan/WPI (20:80) structures. These findings align with those reported by Šeregelj et al. (2021), who investigated the efficient encapsulation of carrot waste extract using WPI and inulin as wall materials, resulting in a powder that achieved approximately 55% encapsulation efficiency for carotenoids through spray drying [16]. Similarly, Ferraz et al. (2022) investigated the co-encapsulation of paprika and cinnamon oleoresin via spray drying, employing WPI and maltodextrin as wall materials, achieving an encapsulation efficiency exceeding 80% [34]. These findings indicate the efficacy of WPI in combination with a polysaccharide for efficiently entrapping carotenoids within the encapsulated structures, thereby highlighting its potential as a promising wall material for carotenoid-encapsulation applications. The maximum EE of β-carotene (%) was noticed at the following experimental conditions: *Χ*_1_ = 6.0% *w*/*w*, *Χ*_2_ = 500 mL/h, *Χ*_3_ = 170 °C.

### 3.2. Fitting Model to Data

The outcomes of fitting the experimental data to the mathematical model (according to Equation (3)) are presented in Table 2. The *R*^2^ value was 0.87 for the EE of SP encapsulants, indicating that the model satisfactorily described the real relationship between the selected parameters. The F-values and *p*-values demonstrate the significance of each coefficient for the different terms; the larger the F-value in comparison to the *p*-value, the more significant is the corresponding response variable [9]. The fitted surface model for SP encapsulants is given in Equation (6).

(6) $$EE=421.50+25.53X_{1}+0.01X_{2}-4.97X_{3}-2.17X_{1}^{2}+0.01X_{3}^{2}+0.02X_{1}X_{3},$$

The variables with the greatest effects on EE were found to be the linear and quadratic terms of the emulsion concentration (*X*_1_, *X*_1_^2^) (*p* < 0.001), followed by the quadratic term of the emulsion flow rate (*X*_2_^2^) (*p* < 0.01), and the linear term (*X*_2_) (*p* < 0.05). In contrast, the linear and quadratic term of the inlet temperature (*X*_3_, *X*_3_^2^) and the interactions between emulsion concentration and emulsion flow rate (*X*_1_*X*_2_), emulsion concentration and inlet temperature (*X*_1_*X*_3_), and emulsion flow rate and inlet temperature (*X*_2_*X*_3_) did not exhibit any significant impact on the EE of SP encapsulants within the range of values studied for each variable (*p* > 0.05). Subsequently, a new regression analysis was conducted, excluding the variables that had no significant effect on EE. The resulting fitted surface model is expressed in Equation (7). The model’s F-value of 6328.04 and the low probability value (*p* < 0.0001) affirm the high reliability of the revised model, signifying its capability to predict the response variable effectively:

(7) $$EE=-17.47+27.15X_{1}+0.03X_{2}-2.07X_{1}^{2}$$

**Figure 1:**
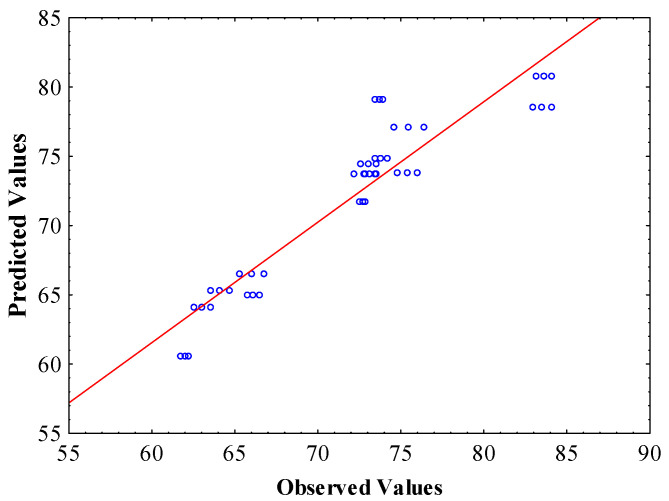
Graph displaying the predicted versus actual values for EE% of SP encapsulants.

Finally, according to Figure 1, it was observed that there was a high concentration and consistent deviation of points around the diagonal (small errors) across the entire range of values examined for the EE of SP encapsulants. Therefore, the model consistently predicted the EE of β-carotene through spray drying.

In summary, the factor that exerted the most significant impact on EE was the concentration of the wall material. This finding is consistent with the studies of Mohammed et al. (2017) and Corrêa-Filho et al. (2019), which reported a positive effect of wall-material concentration on the EE of *Nigella sativa* oil and β-carotene, respectively, whereby increasing the concentration led to a reduction in the core content at the surface of the powder particles [30,35]. This phenomenon may be associated with the shortened duration needed to establish a surface crust on the atomized droplets during the initial drying phase, as the concentration of solids in the feed solution rises. This rapidly forming crust acts as a barrier, preventing the diffusion of core material to the surface of the particles [36].

### 3.3. Three-Dimensional Response Surface Plots for EE of SP

**Figure 2:**
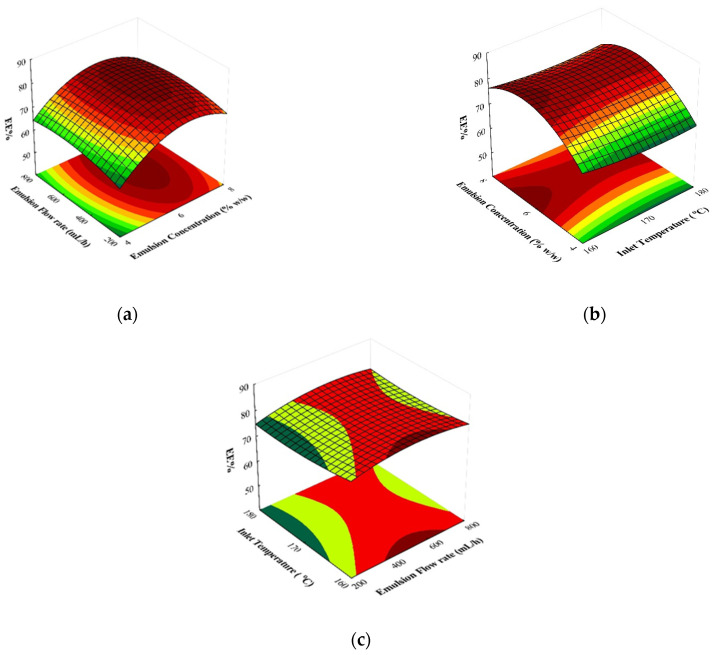
Three-dimensional response surface plots showing the effects of emulsion flow rate, emulsion concentration, and inlet temperature on EE % of SP encapsulants: (**a**) Emulsion flow rate vs. emulsion concentration (inlet temperature: 170 °C); (**b**) emulsion concentration vs. inlet temperature (emulsion flow rate: 500 mL/h); (**c**) inlet temperature vs. emulsion flow rate (emulsion concentration: 6% *w*/*w*).

In Figure 2, the predicted model responses are depicted through three-dimensional response surface plots for the EE of SP encapsulants. Figure 2a illustrates the EE as a function of emulsion flow rate and concentration at a fixed inlet temperature of 170 °C. According to the response surface plots, it was evident that for each emulsion flow rate value, an increase in the concentration of the emulsion wall material initially resulted in an increase in EE from ~65% to ~85%, followed by a subsequent decrease to ~73%. Similar observations were documented by Zahran et al. (2023), who examined the impact of process conditions on the microencapsulation of echium oil via spray drying, suggesting that the content of solids exerted a positive influence on the EE [37]. Figure 2b presents the effects of varying the emulsion concentration and inlet temperature on EE at a fixed emulsion flow rate of 500 mL/h. Similar to Figure 2a, an elevation in the emulsion wall material’s concentration corresponded to an increase in EE for each inlet temperature, underscoring the significant impact of wall-material concentration on EE. Comparable outcomes have also been documented in studies concerning the encapsulation of palm oil [38] and rosemary oil [39]. Finally, Figure 2c shows the interaction between inlet temperature and emulsion flow rate at a fixed emulsion concentration of 6%. As depicted in this figure, an increase in the inlet temperature resulted in a decrease in EE from ~85% to ~74%, possibly due to the thermosensitive nature of β-carotene. Notably, higher inlet temperatures led to increased degradation of β-carotene due to the exposure to heat, which can cause isomerization and oxidation. Results indicated that as the inlet temperature increased, the EE and β-carotene retention decreased. This suggests that lower inlet temperatures are preferable to minimize thermal degradation and enhance the stability of β-carotene in the final product [35]. Furthermore, the reduced efficiency of encapsulation at higher temperatures could be attributed to the temperature’s impact on layer formation and the rate of water evaporation, leading to the breakdown of the crust. This implies that elevated temperatures accelerate the drying of the wall material more rapidly than the core material, resulting in the formation of cracks and pores on the particle surfaces, leading to oil leakage. Similar findings were observed by Tao et al. (2024) and Čulina et al. (2023) in their investigations on the microencapsulation of grapeseed oil and sea buckthorn berry oil, respectively [40,41].

### 3.4. Comparison of EE and Y of SP and FZ Encapsulants

Table 3 presents pullulan/WPI (20:80 *w*/*w*) emulsion properties (stability, viscosity, and droplet size distribution) and the EE of SP and FZ encapsulants with varying wall material concentrations from 4 to 8% *w*/*w*. Based on these results, it is evident that the concentration of the wall material significantly influenced both the emulsion properties and the EE (*p* < 0.05). Specifically, emulsion stability increased at concentrations of 6% and 8% *w*/*w* compared with 4% *w*/*w*. Additionally, the droplet size decreased and the viscosity increased with increasing concentration of wall material. The EE ranged from 64.13 ± 0.59% to 83.54 ± 0.57% for SP encapsulants and from 61.62 ± 0.24% to 70.98 ± 0.87% for FZ encapsulants. The spray-drying and freeze-drying processes both resulted in structures with high β-carotene loads, although spray drying appeared to be more effective in creating β-carotene-loaded structures. Several factors contributed to the superior performance of spray drying. First, the rapid evaporation of the solvent in the hot air chamber during spray drying quickly formed a protective barrier around the β-carotene, minimizing exposure to oxygen and heat, thus reducing degradation and oxidation [42]. Additionally, spray drying typically produces smaller, more uniform particles with a larger surface area, which improves the efficiency of encapsulation and dispersibility in aqueous solutions [35]. Finally, the operational flexibility of spray drying allows precise control over process parameters, enabling optimization for maximum encapsulation efficiency and stability. These factors collectively contributed to the higher encapsulation efficiency and better performance of spray drying in creating β-carotene-loaded structures compared with freeze drying.

The emulsion properties, influenced by the wall material’s concentration, significantly affected EE with both encapsulation techniques (Table 3). For the spray-drying process, increasing the emulsion concentration up to 6% *w*/*w* led to an increase in EE, followed by a decrease at 8% *w*/*w*. Generally, an increase in solid content led to higher EE due to rapid crust formation inhibiting oil diffusion to the particle surface. However, surpassing the optimum concentration limit, which in our system was 6% *w*/*w*, resulted in larger droplet formation, delaying crust formation and leading to β-carotene diffusion to the particle surface [43].

For the freeze-drying process, a higher EE of β-carotene was achieved with increasing emulsion concentration, and the optimal EE was achieved with a wall-material concentration of 8% *w*/*w*. In more detail, increasing the wall-material concentration of emulsions resulted in higher EE values of β-carotene due to the reduction of internal oil circulation and prevention of migration to the surface of the lyophilized structure. This aligns with findings from studies by Fioramonti et al. (2017) and González-Ortega et al. (2020), who demonstrated that increasing the concentration of the wall material positively impacted the EE of flaxseed oil and olive leaf extract, respectively [44,45].

Likewise, Y values for SP ranged from 71.22 ± 0.38% to 73.85 ± 1.39%, while those for FZ-Y ranged from 81.14 ± 0.21% to 84.04 ± 2.56%, with no significant changes among the wall-material concentrations tested (*p* < 0.05). The highest Y value of 84.04% was achieved using the FZ encapsulation process, followed by SP with a value of 73.85%. These Y values align with previous studies, including those for encapsulated *Pulicaria jaubertii* extract (83.16–87.95%) [46] and *Citrus aurantium* essential oil (77.61–90.22%) [47] through freeze drying, and encapsulated sea buckthorn berry oil (64.60%) through spray drying [40]. The lower yield of spray-dried powders can be attributed to the adhesion of feed materials to the walls of the spraying chamber after atomization and the reduced efficiency of the cyclone in collecting fine particles. For industrial production, a product yield of at least 50% is deemed acceptable, as yields below this threshold are not considered viable [48]. Although Y serves as a quantitative measure of the encapsulation process’s performance, EE offers a more precise assessment of the process’s effectiveness. EE takes into account both the quantity and quality of the entrapped bioactive compounds, excluding those present on the surface of the encapsulated structures (not encapsulated). Consequently, a comprehensive evaluation of the encapsulation processes should prioritize encapsulation efficiency before considering the yield results.

Based on the comprehensive analysis of encapsulation efficiency (EE) and considering the pivotal role of wall material concentration, SP encapsulants at 6% *w*/*w* and FZ encapsulants at 8% *w*/*w* of pullulan/WPI (20:80) were subjected to further characterization.

### 3.5. Morphological Characterization of SP and FZ Encapsulants

Morphological properties are crucial features of microencapsulates as they directly influence the stability and release of entrapped active compounds. Table 4 presents SEM images of SP and FZ encapsulants with varying wall material concentrations from 4 to 8% *w*/*w*. The images reveal distinct morphologies resulting from spray drying and freeze drying processes. Specifically, SP encapsulants display a spherical morphology with uneven surfaces, devoid of observable cracks or fractures within the examined range of wall material concentrations. This morphology is desirable, as the absence of pores and cracks, particularly on the surface, prevents the release of entrapped bioactive compounds interacting with the surrounding environment. Additionally, the presence of dents on the surface of SP microencapsulants confirms the rapid formation of a crust before the expansion of droplets, consistent with previous studies on natural pigment encapsulation using pullulan, whey protein isolate, and soy protein via spray drying [49,50]. The average mean diameter of SP encapsulants ranged from 4.04 ± 1.64 μm to 7.19 ± 2.73 μm, indicating a slight, insignificant increase in particle diameter with increasing emulsion concentration (*p* > 0.05). This minor increase can be attributed to the rising viscosity of emulsions as the wall material’s concentration increased. According to Teo et al. (2021), higher viscosity in the feed solution leads to the formation of larger droplets during atomization [51]. For FZ encapsulants, there were no significant differences in the morphology among the samples with varying concentrations of wall material. FZ structures showed an amorphous glass-like formation with a leafy appearance, a characteristic structure of many lyophilized products from aqueous solutions or suspensions [44]. Similar SEM images have also been reported by other researchers for freeze-dried encapsulated pigments in WPI and pullulan [16,52].

### 3.6. ATR-FTIR Analysis of SP and FZ Encapsulants

**Figure 3:**
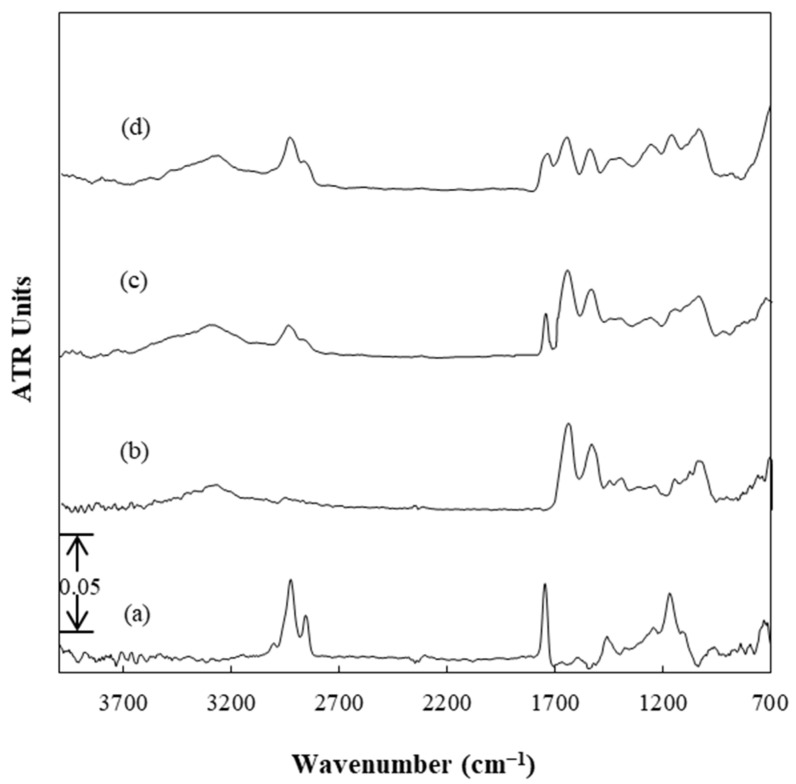
Infrared absorbance spectra of (**a**) β-carotene oil suspension, (**b**) dried pullulan/WPI (20:80 *w*/*w*), (**c**) SP, and (**d**) FZ encapsulants.

ATR-FTIR spectroscopy was used to identify any possible intermolecular interactions between β-carotene and the pullulan/WPI (20:80 *w*/*w*) components as well as to verify the existence of β-carotene in the SP and FZ encapsulated structures. Figure 3 displays the infrared spectra of β-carotene oil suspension, dried pullulan/WPI (20:80 *w*/*w*) structures, and dried SP and FZ encapsulants. The presence of β-carotene was indirectly confirmed through analyzing the infrared spectra of the structures, as evidenced by the characteristic peaks of its dissolution medium (corn oil), due to its low mass ratio relative to the pullulan/WPI components and the oil present in the structures. Specifically, the spectra of SP and FZ encapsulants exhibited characteristic peaks of both the carrier material and the oil containing the β-carotene. The dried pullulan/WPI (20:80 *w*/*w*) structures without β-carotene displayed characteristic bands of their polymeric components, as described in detail in previous studies [9,26]. Moreover, in the spectra of SP and FZ encapsulants, increased absorption was observed in the wavenumber range between 3000 cm^−1^ and 2800 cm^−1^ compared with the unloaded counterparts due to the presence of oil in the structures. Additionally, a peak at 1747 cm^−1^ corresponding to the C=O groups of oil triglycerides was recorded, confirming the successful retention of the active substance within the mixed polymeric carrier [53]. Overall, based on the ATR-FTIR results, β-carotene was effectively integrated into the SP and FZ encapsulants. Importantly, the absence of new peaks or shifts in existing ones indicated that the encapsulated substance retention occurred solely through intermolecular interactions without involving chemical reactions.

### 3.7. Water Adsorption Behavior

**Figure 4:**
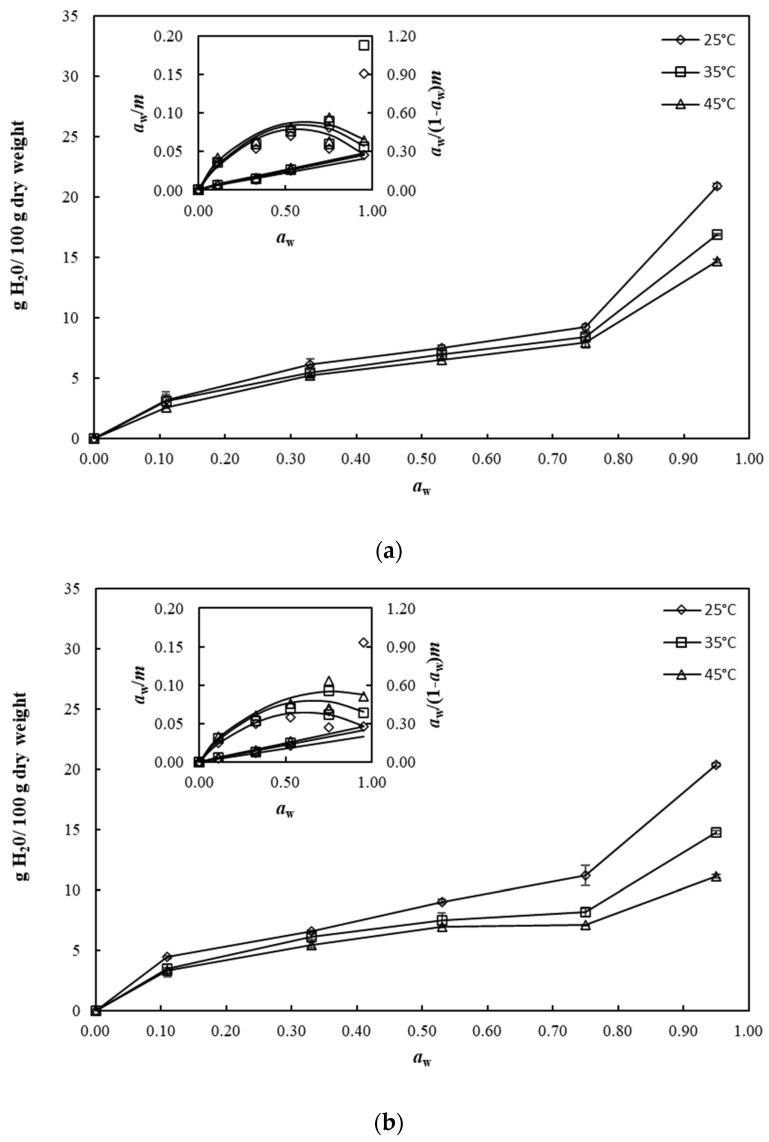
Water adsorption isotherms for (**a**) SP and (**b**) FZ encapsulants at varying water-activity levels and temperatures. The associated GAB and BET plots are provided in the insets for reference.

The experimental moisture sorption data of SP and FZ encapsulants are displayed in Figure 4a and Figure 4b, respectively, at three different temperatures: 25 °C, 35 °C, 45 °C. The adsorption isotherms of the examined samples exhibited a sigmoidal shape, characteristic of high molecular weight biopolymers and foods rich in polysaccharides and proteins [54]. Based on the adsorption isotherms, it was observed that at constant temperature, there was an increase in the equilibrium moisture of the samples with a corresponding increase in *a*_w_. Conversely, at constant *a*_w_, the moisture content decreased with increasing temperature, indicating that the microencapsulated β-carotene powder became less hygroscopic at higher temperatures. Notably, at lower temperatures, proteins and carbohydrates exhibit a higher water binding capacity, while at higher temperatures, there is increased breaking of hydrogen bonds or separation of water molecules [55]. FZ encapsulants exhibited slightly higher moisture content compared with SP ones, probably attributable to the greater pore formation during the FZ process, enabling enhanced moisture absorption into the microcapsules’ pores.

The experimental moisture sorption data of SP and FZ encapsulants were fitted to the BET and GAB adsorption models within the range of *a*_w_ 0.11–0.64 and 0.11–0.95, respectively, and the depictions of models are presented in the inset in Figure 4. According to the results, both models quite well describe the experimental data for SP and FZ encapsulants. In more detail, the GAB model provided a satisfactory fit for the adsorption data at all temperatures and across the entire range of *a*_w_ levels investigated. In contrast, BET model was suitable for describing the adsorption data only within the range of water activity from 0.11 to 0.64. Comparable observations regarding the fitting of sorption models such as BET and GAB have been reported by various researchers investigating the sorption isotherms of encapsulated carotenoids and pigments using spray-drying and freeze-drying methods [56,57]. The parameter values of the BET and GAB models for SP and FZ encapsulants are presented in Table 5. One of the crucial parameters is the monolayer value (*m*_m_), which represents the quantity of water strongly adsorbed at particular sites within the material, which is deemed significant for ensuring the stability of food products. The *m*_m_ represents a crucial moisture threshold that maximizes the storage time while minimizing product quality deterioration at a specified temperature. In the present study, the estimated *m*_m_ values fell within the range of 3.35–3.85 and 3.44–4.88 g H_2_O/100 g dry weight, for SP and FZ encapsulants, respectively, depending on the equation used for estimation and the storage temperature. FZ encapsulants showed higher values of *m*_m_ than SP ones at all storage temperatures, possible attributed to the differences in the morphology of structures resulting from the spray-drying and freeze-drying processes, indicating greater availability of specific water-adsorption sites. Furthermore, for both samples, *m*_m_ values showed a slightly decreasing trend with increasing storage temperature, possibly due to the reduction in the number of active binding sites exposed as a result of physicochemical changes caused by the temperature change [58]. Similarly, the *K*′ and *C* values of the GAB equation decreased with increasing temperature, as reported in a similar study [59].

### 3.8. Glass Transition Temperature

**Figure 5:**
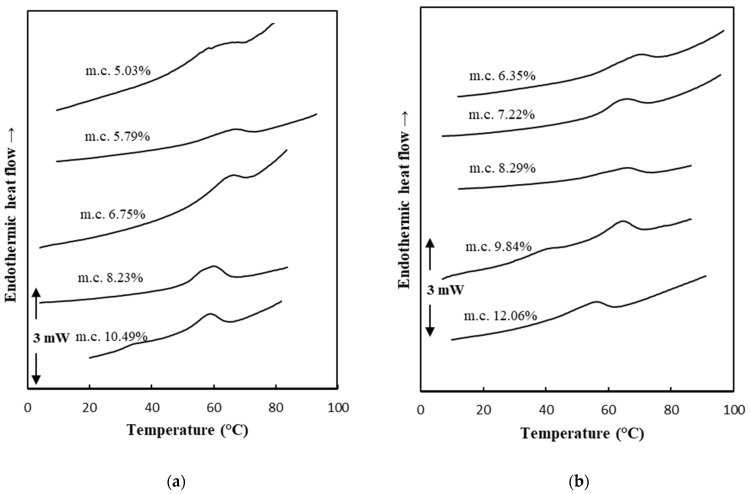
DSC plots during the first heating cycle of (**a**) SP and (**b**) FZ encapsulants equilibrated at varying water-activity levels at 25 °C. The moisture content (m.c. %) is indicated for each sample.

**Figure 6:**
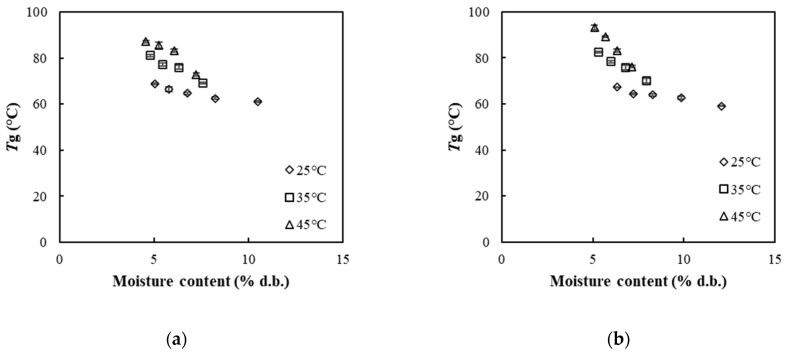
Glass transition temperature (*T*_g_) of (**a**) SP and (**b**) FZ encapsulants as a function of moisture content at different storage temperatures (25, 35, 45 °C).

Figure 5a,b display representative DSC scans for SP and FZ encapsulant samples, respectively, upon their equilibration in different *a*_w_s at 25 °C at the first heating scan. The incorporation of pullulan into the encapsulation structures resulted in the detection of an endothermic transition peak during the first heating cycle of the samples. Such endothermic transitions reflect an enthalpy relaxation mechanism observed in the region of the glass transition temperature zone during the heating of glassy materials [26]. According to these figures, the position of the endothermic peak shifted towards lower temperatures with increasing moisture content in the samples. In Figure 6a,b, *T*_g_ values are presented as a function of the moisture content of SP and FZ encapsulants at three different temperatures (25, 35, 45 °C), respectively. For SP encapsulants, *T*_g_ values ranged from 61.01 °C to 68.79 °C, from 69.15 °C to 81.10 °C, and from 72.82 °C to 87.17 °C at the three different temperatures (25, 35, 45 °C), respectively. Similarly, for FZ encapsulants, *T*_g_ values ranged from 59.01 °C to 67.41 °C, from 70.13 °C to 82.45 °C, and from 76.28 °C to 93.25 °C at the three different temperatures (25, 35, 45 °C), respectively. The reported *T*_g_ values align with the existing literature on WPI samples or WPI blends with polysaccharides within similar ranges of water content [60]. Based on the results, as expected, an increase in moisture content corresponded to lower *T*_g_ values, attributed to water acting as a plasticizer for the carrier’s amorphous matrix. Furthermore, higher storage temperatures were associated with higher *T*_g_ values [9].

### 3.9. Storage Stability of SP and FZ Encapsulants under Different a_w_ and Temperature Conditions

**Figure 7:**
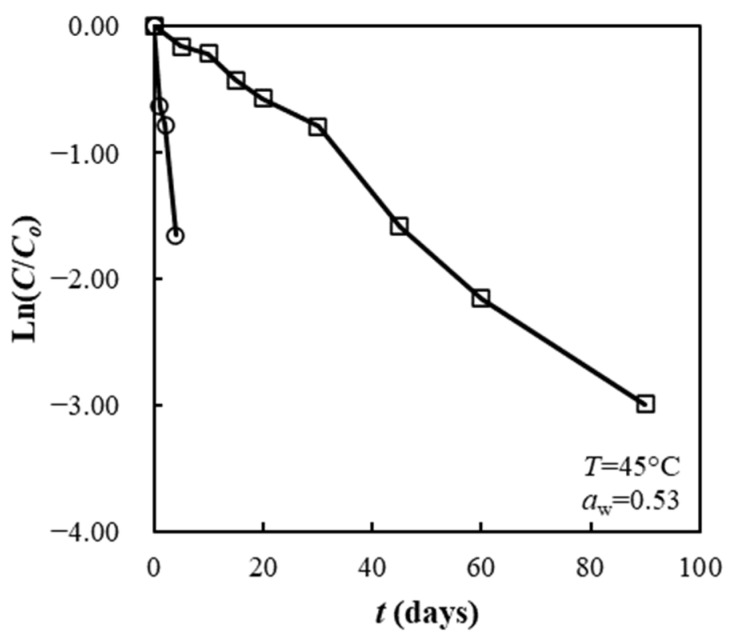
First-order degradation kinetic plots for SP (squares) and FZ (circles) encapsulants.

Figure 7 presents an illustrative example of degradation data kinetics plots for SP and FZ encapsulants stored at 45 °C and *a*_w_ = 0.53. For both of the encapsulation structures studied, the plots of Ln(*C*/*C*_o_) against time displayed a linear relationship, indicating first-order degradation kinetics, except for the samples stored in water activity of 0.11 and 0.33 at 25 °C. In these cases, a rapid decrease in the *C*/*C*_o_ values occurred during the first five days of storage, followed by a reduction in the degradation rate over time. This phenomenon was attributed to the initial rapid degradation of β-carotene located near the surface of the particulate carriers, followed by a decrease in oxygen diffusion inside the structures, allowing oxidative degradation to continue.

Table 6 reports the kinetic parameters of the SP and FZ encapsulants, including the degradation rate constant (*k*), the half-life periods (*t*_1/2_), and the coefficients of determination, during their storage at five different *a*_w_ levels (0.11, 0.33, 0.53, 0.75, 0.95) and three different temperatures (25, 35, 45 °C). For SP encapsulants, it was evident that the development of these structures led to decreased constant rates of β-carotene degradation, especially during storage at low water-activity levels (*a_w_* = 0.11 and 0.33). The results indicated that increasing water-activity levels and storage temperature resulted in an increase in the degradation constant rate and a corresponding decrease in the half-life period of β-carotene (*p* < 0.05). Specifically, the increase in *a*_w_ resulted in higher moisture content in the microencapsulated particles, allowing greater water mobility and dissolution of wall materials, thereby facilitating the degradation of the β-carotene [19]. The maximum degradation constant was observed during the storage of microencapsulated β-carotene at intermediate water activity of 0.53. For higher *a*_w_ levels (0.75 and 0.95), the degradation constant rates were lower than those for *a*_w_ = 0.53, but always higher than those in low-water-activity environments (*a*_w_ = 0.11, 0.33). This is because the water under those storage conditions had a protective effect on the oxidation reaction of β-carotene via reducing oxygen diffusion [61]. Therefore, the degradation of β-carotene was influenced by different degradation or protection mechanisms at various moisture levels. For the FZ encapsulants, the degradation of encapsulated β-carotene followed a similar trend to the SP encapsulants during storage at various levels of *a*_w_. This is likely to have been due to the similar composition of the encapsulation matrix used in both cases. Specifically, an increase in degradation constant rate values was observed with increasing *a*_w_ levels. The maximum value of k was recorded at *a*_w_ = 0.53, while reduced values were observed for *a*_w_ = 0.75 and 0.95 compared with the intermediate *a*_w_ level. Similar observations have also been reported by many researchers studying the degradation kinetics of encapsulated carotenoids/colorants through freeze drying. Notably, Mahfoudhi and Hamdi (2015) and Sutter et al. (2007) studied the encapsulation of β-carotene in mannitol and gum arabic/almond gum, respectively, through freeze drying. Their findings indicated that the degradation rate of encapsulated β-carotene increased during storage at low levels of relative humidity [62,63].

Comparing SP and FZ encapsulants in terms of their effectiveness in preserving β-carotene, it can be concluded that FZ structures were less effective in protecting β-carotene under the same storage conditions. As SP and FZ structures are characterized by the same composition, the difference in β-carotene stability can be attributed to the structures of the lyophilized products, which exhibit intense porous formations. The degradation of β-carotene, and non-polar carotenoids in general, depends on the mobility of reactants (e.g., oxygen diffusion through the matrix), which, in turn, is influenced by the macrostructure/ porosity of the matrix, the storage temperature relative to the glass transition temperature of the polymer network, and the thermally dependent physical state of the carrier. Therefore, the presence of micropores increases the contact area, allowing oxygen diffusion through the matrix, reducing the oxidative stability of lyophilized products. Similar conclusions were reached by Haas et al. (2019), who studied the stability of encapsulated carotenoid structures from carrots and fish oil/limonene using spray-drying and freeze-drying techniques [64]. The porous structure of the lyophilized products led to the rapid degradation of carotenoids due to the increased exposure of active ingredients to environmental factors. Therefore, freeze-drying does not appear to be the most suitable technique for long-term storage of encapsulated β-carotene.

### 3.10. Degradation Rates in Relation to Storage Temperature

The impact of storage temperature on the degradation of SP and FZ encapsulants was investigated at three distinct temperatures: 25, 35, and 45 °C. The findings revealed a notable disparity in the degradation rate of β-carotene at these three storage temperatures for each tested level of water activity (*p* < 0.05). At 25 °C, greater stability of β-carotene was observed compared with the other storage temperatures, due to the increased interactions between the structures and water vapors, which hindered the diffusion of oxygen and thus allowed its preservation in the encapsulation structures. Furthermore, in general, increasing the temperature accelerates chemical processes due to the increased kinetic energy of the reactants. Similar results were reported in the study of Kha et al. (2015), who investigated the degradation kinetics of encapsulated carotenoid oils in gac (*Momordica cochinchinensis*) plants using spray drying at various storage temperatures [65]. Additionally, similar findings were also obtained in studies on the encapsulation of β-carotene in starch, fucoxanthin in maltodextrin, and lycopene from tomato by-products in gelatin and polyglutamic acid using freeze drying [66,67,68].

**Figure 8:**
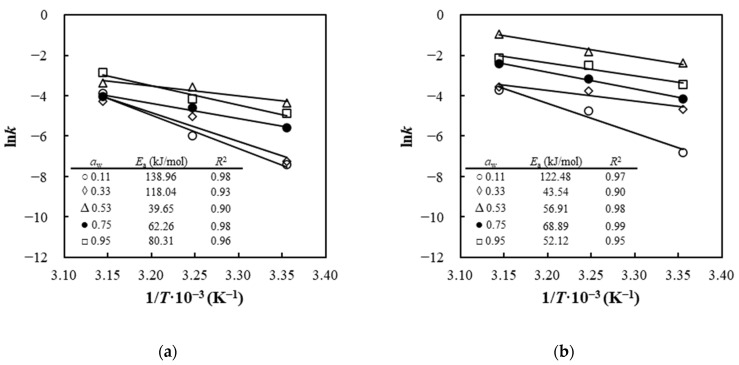
Arrhenius plots of degradation rate constants for (**a**) SP and (**b**) FZ encapsulants stored at different temperatures and water-activity levels.

The impact of storage temperature on the degradation of SP and FZ encapsulants was expressed through fitting the data of the constant rates to the Arrhenius model for each *a*_w_ level at the three different temperatures (25, 35, 45 °C). The graph of the experimental data ln*k* as a function of 1/*T* for SP and FZ encapsulants is shown in Figure 8a and Figure 8b, respectively, demonstrating separate lines for each *a*_w_ level. For all samples, the Arrhenius model exhibited excellent fits (*R*^2^ > 0.90) for all water activity levels studied. Using linear regression analysis of the slopes of the lines in Figure 8, the activation energy (*E*_a_) for the degradation of SP and FZ encapsulants was determined. For SP and FZ encapsulants, *E*_a_ ranged from 39.65 to 138.96 kJ/mol and from 43.54 to 122.48 kJ/mol, respectively, with higher values observed at lower water activities, indicating a greater temperature sensitivity in the degradation of β-carotene at low *a*_w_ values. This phenomenon arises from alterations in the structure of encapsulants caused by water plasticization. In particular, the collapse of the wall matrix due to significant water sorption on the polar sites of the biopolymers can contract the micropores, thereby influencing the diffusion of reactants and subsequently regulating the rate of oxidation. Consequently, the lower *E*_a_ at high *a*_w_ values is ascribed to variations in the macrostructure and porosity of the encapsulants, which reduce the rate of oxygen diffusion through the wall material, thus protecting the β-carotene. A similar trend was observed by Sarpong et al. (2019), who reported that the *E*_a_ for the degradation of β-carotene was observed to exhibit a slight dependence on the *a*_w_ level, with values ranging from approximately 16 to 18 kJ/mol at *a*_w_ levels of 0.10 and 0.30, respectively [69].

### 3.11. Photostability of SP and FZ Encapsulants

**Figure 9:**
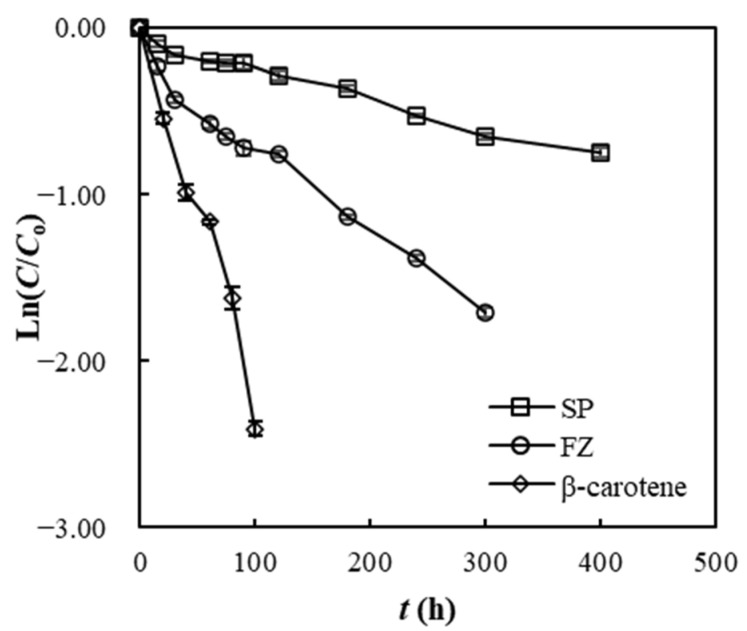
Photodegradation plots for free β-carotene, SP, and FZ encapsulants.

The kinetic plots of photodegradation data for free β-carotene, SP, and FZ encapsulants are depicted in Figure 9. Consistent with the degradation kinetics observed at various water activity levels and temperatures, the UV–Vis light treatment data were modeled using a first-order reaction model, resulting in excellent fits with *R*^2^ values surpassing 0.97. Table 7 displays the photodegradation rate constants (*k*) and half-life period values for SP and FZ encapsulants.

According to the findings, SP and FZ encapsulants extended the half-life of β-carotene 11- and 4-fold, respectively, compared with free β-carotene. Therefore, both structures demonstrated their effectiveness in protecting β-carotene during UV–Vis irradiation exposure. Similar studies have utilized carriers such as maltodextrin, modified starch, and gum arabic for the encapsulation of curcumin via spray drying, preventing its oxidation loss during light exposure [70]. Comparable conclusions have also been reported for the encapsulation of astaxanthin in gum arabic and maltodextrin [71], canthaxanthin in soybean polysaccharide [72], and lycopene in sucrose and gelatin [73]. Furthermore, similar studies have used carriers such as gelatin and polyglutamic acid to encapsulate lycopene from tomato by-products, as well as gum arabic for encapsulating curcumin through freeze drying [68,70].

Comparing SP and FZ encapsulants, SP ones seem to be more effective in delaying the photo-oxidation of β-carotene. This can be attributed to the SP’s morphology, characterized by a uniform structure without cracks and breakage, led to the optimal preservation of the encapsulated β-carotene. In contrast, the porous structure of the FZ samples resulted in greater exposure of β-carotene to UV–Vis irradiation. Similar conclusions were reported by Cano-Higuita et al. (2015), who compared the encapsulation structures of curcumin in a mixture of gum arabic, modified starch, and maltodextrin applied through spray drying and freeze drying. They reported that the encapsulation structures developed through spray drying exhibited higher curcumin retention rates after 8 weeks of exposure to light [70].

Overall, to enhance the stability and reduce the degradation of β-carotene encapsulates during storage, several measures can be implemented. Firstly, storing encapsulated β-carotene in low humidity and at temperatures below 25 °C can significantly minimize thermal degradation and oxidative damage. Utilizing packaging materials with high oxygen barrier properties, such as metallized films or multi-layered plastic containers, can further protect β-carotene from exposure to oxygen and light, which are primary factors contributing to its degradation. Adding antioxidants such as ascorbic acid or tocopherols to the encapsulation matrix can provide additional protection via scavenging free radicals and inhibiting oxidative reactions. Additionally, employing vacuum or inert gas flushing during the packaging process can reduce oxidative stress through eliminating residual oxygen. These combined strategies can significantly enhance the shelf life and efficacy of β-carotene encapsulates, ensuring their stability and functionality in various applications.

## 4. Conclusions

In this study, β-carotene was efficiently encapsulated using pullulan and WPI as a composite matrix at a weight ratio of 20:80, employing both spray-drying and freeze-drying techniques, yielding high encapsulation efficiencies (EE > 60%). The influence of processing parameters such as wall-material concentration, flow rate, and inlet temperature on the performance of SP encapsulants, as well as wall-material concentration on that of FZ encapsulants, was examined. Increasing the concentration of the wall material had the greatest impact on the EE of both types of encapsulants, leading to higher levels of EE. SP encapsulants exhibited a spherical morphology with uneven surfaces and no observable cracks or fractures, while FZ structures showed an amorphous glass-like formation with a leafy appearance. Concerning the encapsulation efficiency, SP yielded higher encapsulation efficiencies compared with FZ, indicating that SP encapsulants were more effective in creating β-carotene-loaded structures. Furthermore, SP encapsulants provided greater protection than FZ to β-carotene against oxidative degradation under different storage temperatures, *a*_w_ levels, and upon exposure to UV-Vis irradiation. This difference in effectiveness may be attributed to the presence of micropores that increased the contact area and allowed oxygen diffusion through the matrix, thereby reducing the oxidative stability of the lyophilized products. Both types of encapsulants exhibited enhanced stability in low-humidity conditions, with the peak values of the degradation rate constants for β-carotene observed at an intermediate level of water activity (*a*_w_ = 0.53) across all three storage temperatures investigated. The observed differences in *E*_a_ at different *a*_w_ levels were influenced by the structural characteristics of the encapsulation matrices, suggesting greater temperature sensitivity in the degradation of β-carotene at low water-activity values. These variations in macrostructure and porosity contributed significantly to the rate of oxygen diffusion and subsequent protection of β-carotene. Overall, the results of the present study may open up new opportunities in the utilization of SP encapsulants with potential applications for functional foods, pharmaceuticals, and carotenoid supplements.

Future research will focus on testing other encapsulation matrices to enhance the stability and bioavailability of β-carotene and other bioactive compounds. Different combinations of natural polymers and proteins will be explored to gain insights into optimizing encapsulation efficiency and protecting sensitive ingredients. Additionally, applying optimized encapsulation techniques in real food systems will serve to evaluate the practical applicability and effectiveness of these encapsulated ingredients in various food products. This will involve studying the interactions between encapsulated bioactives and food matrices, as well as assessing their sensory, nutritional, and functional properties in final food formulations. Moreover, investigating the gastrointestinal release kinetics of the encapsulated compounds will be crucial to ensuring their bioavailability and efficacy upon consumption. This comprehensive approach will help in understanding the full potential of these encapsulation strategies in enhancing the stability and functionality of bioactive ingredients in food applications.

## Figures and Tables

**Table 1:** Encapsulation efficiency (EE) (dependent variable) responses of SP encapsulants based on central composite design.

	Variable Levels			Observed Values
Run	*X*_1_ (Emulsion Concentration, % *w*/*w*)	*X*_2_ (Emulsion Flow Rate, mL/h)	*X*_3_ (Inlet Temperature, °C)	EE (%)
1	4	200	160	63.07 ± 0.50 ^g^
2	4	800	160	66.02 ± 0.74 ^e^
3	4	500	170	64.13 ± 0.59 ^f^
4	4	200	180	62.00 ± 0.25 ^h^
5	4	800	180	66.14 ± 0.34 ^e^
6	6	500	160	83.62 ± 0.47 ^a^
7	6	200	170	73.82 ± 0.36 ^c^
8	6	500	170	83.54 ± 0.57 ^a^
9	6	500	170	83.54 ± 0.57 ^a^
10	6	800	170	75.50 ± 0.89 ^b^
11	6	500	180	73.70 ± 0.26 ^c^
12	8	200	160	73.12 ± 0.35 ^c,d^
13	8	800	160	72.87 ± 0.65 ^c,d^
14	8	500	170	73.08 ± 0.47 ^c,d^
15	8	200	180	72.72 ± 0.16 ^d^
16	8	800	180	75.42 ± 0.62 ^b^

Values labeled with different letters indicate significant differences (*p* < 0.05).

**Table 2:** Analysis of variance for the response surface EE for SP encapsulants.

Source	Coefficients	Standard Error	Sum of Squares	DF	Mean Square	F-Value
Model	421.50	271.47	234,345.29	10	23,434.53	3407.19 ***
*X*_1_	25.53	5.38	630.65	1	630.65	91.69 ***
*X*_2_	0.01	0.03	37.75	1	37.75	5.49 *
*X*_3_	−4.97	3.22	22.73	1	22.73	3.30 ^NS^
*X*_1_^2^	−2.17	0.24	580.98	1	580.98	84.47 ***
*X*_2_^2^	0.00	0.00	53.28	1	53.28	7.75 **
*X*_3_^2^	0.01	0.01	14.61	1	14.61	2.12 ^NS^
*X*_1_ *X*_2_	0.00	0.00	8.10	1	8.10	1.18 ^NS^
*X*_1_ *X*_3_	0.02	0.03	3.57	1	3.57	0.52 ^NS^
*X*_2_ *X*_3_	0.00	0.00	6.44	1	6.44	0.94 ^NS^
Residual			240.73	35	6.88	
Total			1849.09	44		

* *p* < 0.05, ** *p* < 0.01, *** *p* < 0.001, NS—Not Significant. The coefficient of determination (*R*^2^) of model was 0.87.

**Table 3:** Effect of wall-material concentration on the emulsion properties (stability, viscosity, and droplet size distribution) and EE of SP (process conditions: 500 mL/h, 170 °C) and FZ encapsulants.

Wall-Material Concentration (% *w*/*w*)	*ESI* (%)	*d*_4,3_ (μm)	Viscosity (mPa s)	SP-EE%	FZ-EE%	SP-Y%	FZ-Y%
4	97.6 *±* 0.26 ^a^	1.82 *±* 0.04 ^a^	36.20 *±* 1.13 ^a^	64.13 *±* 0.59 ^a^	61.62 *±* 0.24 ^c^	71.22 ^a^ *±* 0.38	81.14 ^a^ *±* 0.21
6	100 *±* 0.00 ^b^	1.75 *±* 0.12 ^a^	90.30 *±* 1.27 ^b^	83.54 *±* 0.57 ^b^	66.48 *±* 0.63 ^b^	72.39 ^a^ *±* 0.87	82.48 ^a^ *±* 1.46
8	100 *±* 0.00 ^b^	1.58 *±* 0.06 ^b^	435.80 *±* 1.26 ^c^	73.08 *±* 0.47 ^c^	70.98 *±* 0.87 ^a^	73.85 ^a^ *±* 1.39	84.04 ^a^ *±* 2.56

Values labeled with different letters indicate significant differences (*p* < 0.05).

**Table 4:** SEM images of SP (process conditions: 500 mL/h, 170 °C) and FZ encapsulants. Scale bar: 10 μm (SP) and 500 μm (FZ).

Emulsion Concentration (% *w*/*w*)	SP	FZ	Main Characteristics
4	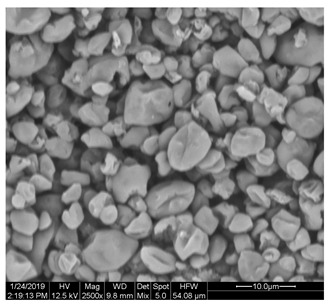	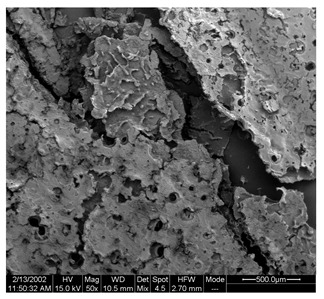	Mean diameter: 4.04 ± 1.64 μm, smooth, spherical particles with no cracks (SP); porous, amorphous structure (FZ).
6	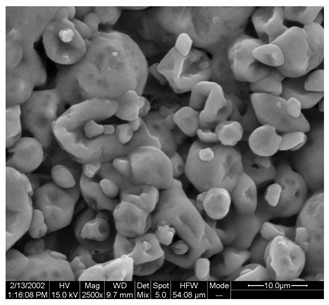	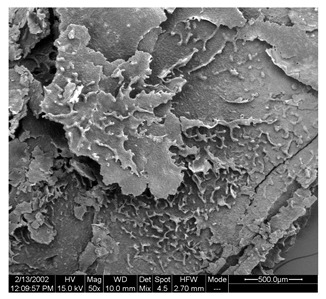	Mean diameter: 5.67 ± 2.02 μm, slight increase in particle size, maintained structural integrity (SP); maintained porous, amorphous structure (FZ).
8	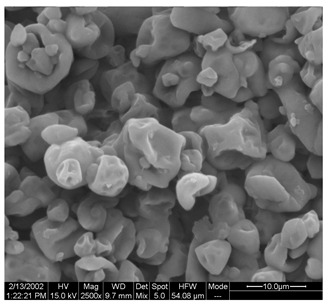	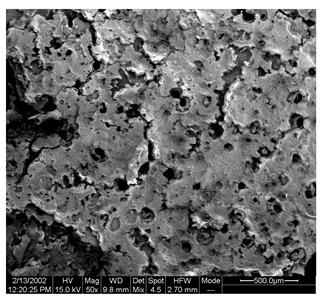	Mean diameter: 7.19 ± 2.73 μm, larger particles with occasional surface dents (SP); maintained porous, amorphous structure (FZ)

**Table 5:** Predictions of the BET and GAB parameters for SP and FZ encapsulants.

	*BET* (*a*_w_ *=* 0.11–0.64)			*GAB* (*a*_w_ = 0.11–0.94)			
Temperature/ Encapsulation Technique	*m_m_* (g H_2_O per 100 g Dry Weight)	*K*	*R^2^*	*m_m_* (g H_2_O per 100 g Dry Weight)	*K*′	*C*	*R* ^2^
25 °C							
SP	3.85	31.28	0.94	3.72	0.86	76.84	0.99
FZ	4.75	78.67	0.99	4.88	0.80	62.66	0.99
35 °C							
SP	3.50	38.84	0.95	3.65	0.82	57.00	0.99
FZ	3.78	52.90	0.94	4.23	0.75	55.09	0.97
45 °C							
SP	3.35	24.53	0.96	3.52	0.79	28.25	0.99
FZ	3.44	64.35	0.96	4.46	0.63	36.84	0.98

**Table 6:** Degradation constant rates (*k*)^a^, half-life periods (*t*_1/2_) and correlation coefficients (in parentheses) for SP and FZ encapsulants stored at different temperatures and water-activity levels.

	*k* × 10^−3^ ± *s*_k_ × 10^−3^ (day^−1^)				
	Half-Life Period *t*_1/2_ (days)				
Samples	0.11	0.33	0.53	0.75	0.94
25 °C					
SP	0.6 ± 0.47 ^1,a^	0.7 ± 0.69 ^1,a^	12.8 ± 1.36 ^1,d^	3.7 ± 0.65 ^1,b^	7.6 ± 1.58 ^1,c^
	911.91	713.21	37.84	181.26	89.94
	(0.94)	(0.89)	(0.99)	(0.95)	(0.99)
FZ	1.1 ± 0.58 ^1,a^	9.5 ± 0.42 ^2,b^	92.8 ± 5.69 ^2,e^	15.8 ± 3.57 ^2,c^	32.3 ± 2.58 ^2,d^
	130.77	58.08	8.92	33.47	17.16
	(0.72)	(0.98)	(0.98)	(0.93)	(0.97)
35 °C					
SP	2.5 ± 0.86 ^1,a^	6.6 ± 1.32 ^1,a,b^	28.7 ± 2.09 ^1,d^	10 ± 2.97 ^1,b^	15.6 ± 3.98 ^1,c^
	276.18	103.63	24.86	71.97	44.78
	(0.99)	(0.99)	(0.97)	(0.98)	(0.98)
FZ	8.6 ± 1.29 ^2,a^	22.9 ± 2.95 ^2,b^	165.1 ± 10.39 ^2,e^	43.1 ± 2.89 ^2,c^	82.7 ± 4.36 ^2,d^
	77.23	30.52	4.30	15.47	7.69
	(0.94)	(0.98)	(0.99)	(0.97)	(0.96)
45 °C					
SP	20.6 ± 2.36 ^1,b^	13.8 ± 1.78 ^1,a^	34.8 ± 5.36 ^1,c^	17.9 ± 2.36 ^1,a,b^	58.8 ± 3.84 ^1,d^
	39.63	45.50	17.88	48.06	9.11
	(0.98)	(0.99)	(0.99)	(0.97)	(0.97)
FZ	24.4 ± 1.84 ^1,a^	28.5 ± 1.91 ^2,a^	395.0 ± 13.69 ^2,d^	90.6 ± 7.37 ^2,b^	120.6 ± 9.54 ^2,c^
	13.56	29.85	1.95	8.35	7.06
	(0.89)	(0.96)	(0.97)	(0.99)	(0.96)

Different superscript numbers indicate significant differences among *k* values for SP and FZ encapsulants at a specified *a*_w_ environment and temperature (*p* < 0.05). Different superscript letters show significant differences among *k* values for the same encapsulation method and temperature in different *a_w_* environments (*p* < 0.05).

**Table 7:** Photo-oxidation constant rates (*k*), half-life periods (*t*_1/2_), and correlation coefficients (*R*^2^) for free β-carotene, SP, and FZ encapsulants.

Sample	*k*·10^−3^ ± *s*_k_·10^−3^ (h^−1^)	*t*_1/2_ (h)	*R* ^2^
β-carotene	22.1 ± 1.49 ^c^	30.52	0.97
SP	1.90 ± 0.23 ^a^	336.02	0.98
FZ	5.30 ± 0.96 ^b^	102.44	0.98

Values labeled with different letters indicate significant differences (*p* < 0.05).

## Data Availability

The data presented in this study are available on request from the corresponding author (privacy reasons).

## References

[1] Garza-Cadena C., Ortega-Rivera D.M., Machorro-García G., Gonzalez-Zermeño E.M., Homma-Dueñas D., Plata-Gryl M., Castro-Muñoz R. (2023). A comprehensive review on Ginger (Zingiber officinale) as a potential source of nutraceuticals for food formulations: Towards the polishing of gingerol and other present biomolecules. Food Chem..

[2] Castro-Muñoz R., Correa-Delgado M., Córdova-Almeida R., Lara-Nava D., Chávez-Muñoz M., Velásquez-Chávez V.F., Hernández-Torres C.E., Gontarek-Castro E., Ahmad M.Z. (2022). Natural sweeteners: Sources, extraction and current uses in foods and food industries. Food Chem..

[3] Meléndez-Martínez A.J., Mandić A.I., Bantis F., Böhm V., Borge G.I.A., Brnčić M., Bysted A., Cano M.P., Dias M.G., Elgersma A. (2022). A comprehensive review on carotenoids in foods and feeds: Status quo, applications, patents, and research needs. Crit. Rev. Food Sci. Nutr..

[4] Saini R.K., Prasad P., Lokesh V., Shang X., Shin J., Keum Y.-S., Lee J.-H. (2022). Carotenoids: Dietary Sources, Extraction, Encapsulation, Bioavailability, and Health Benefits—A Review of Recent Advancements. Antioxidants.

[5] Gul K., Tak A., Singh A.K., Singh P., Yousuf B., Wani A.A. (2015). Chemistry, encapsulation, and health benefits of β-carotene—A review. Cogent Food Agric..

[6] Akram S., Mushtaq M., Waheed A., Mushtaq M., Anwar F. (2021). Chapter 1—β-Carotene: Beyond provitamin A. A Centum of Valuable Plant Bioactives.

[7] Ge W., Li D., Chen M., Wang X., Liu S., Sun R. (2015). Characterization and antioxidant activity of β-carotene loaded chitosan-graft-poly(lactide) nanomicelles. Carbohydr. Polym..

[8] Eriksen N. (2016). Research Trends in the Dominating Microalgal Pigments, β-carotene, Astaxanthin, and Phycocyanin Used in Feed, in Foods, and in Health Applications. J. Nutr. Food Sci..

[9] Drosou C., Krokida M., Biliaderis C.G. (2022). Encapsulation of β-carotene into food-grade nanofibers via coaxial electrospinning of hydrocolloids: Enhancement of oxidative stability and photoprotection. Food Hydrocoll.

[10] Eun J.-B., Maruf A., Das P.R., Nam S.-H. (2020). A review of encapsulation of carotenoids using spray drying and freeze drying. Crit. Rev. Food Sci. Nutr..

[11] Elik A., Koçak Yanık D., Göğüş F. (2021). A comparative study of encapsulation of carotenoid enriched-flaxseed oil and flaxseed oil by spray freeze-drying and spray drying techniques. LWT.

[12] Santos P.D.d.F., Rubio F.T.V., Balieiro J.C.d.C., Thomazini M., Favaro-Trindade C.S. (2021). Application of spray drying for production of microparticles containing the carotenoid-rich tucumã oil (*Astrocaryum vulgare* Mart.). LWT.

[13] Fang S., Zhao X., Liu Y., Liang X., Yang Y. (2019). Fabricating multilayer emulsions by using OSA starch and chitosan suitable for spray drying: Application in the encapsulation of β-carotene. Food Hydrocoll..

[14] Lim A.S.L., Roos Y.H. (2016). Spray drying of high hydrophilic solids emulsions with layered interface and trehalose-maltodextrin as glass formers for carotenoids stabilization. J. Food Eng..

[15] Sansone F., Esposito T., Mencherini T., Del Prete F., Cannoniere A.L., Aquino R.P. (2023). Exploring microencapsulation potential: Multicomponent spray dried delivery systems for improvement of Chlorella vulgaris extract preservation and solubility. Powder Technol..

[16] Šeregelj V., Ćetković G., Čanadanović-Brunet J., Šaponjac V.T., Vulić J., Lević S., Nedović V., Brandolini A., Hidalgo A. (2021). Encapsulation of carrot waste extract by freeze and spray drying techniques: An optimization study. LWT.

[17] Coelho L.M., Gonçalves I., Ferreira P., Pinheiro A.C., Vicente A.A., Martins J.T. (2022). Exploring the performance of amaranth grain starch and protein microcapsules as β-carotene carrier systems for food applications. Food Struct..

[18] Cao L., Xu Q., Xing Y., Guo X., Li W., Cai Y. (2020). Effect of skimmed milk powder concentrations on the biological characteristics of microencapsulated Saccharomyces cerevisiae by vacuum-spray-freeze-drying. Dry. Technol..

[19] Chen J., Li F., Li Z., McClements D.J., Xiao H. (2017). Encapsulation of carotenoids in emulsion-based delivery systems: Enhancement of β-carotene water-dispersibility and chemical stability. Food Hydrocoll..

[20] Constantino A.B.T., Garcia-Rojas E.E. (2023). Microencapsulation of beta-carotene by complex coacervation using amaranth carboxymethyl starch and lactoferrin for application in gummy candies. Food Hydrocoll..

[21] Lim A.S.L., Burdikova Z., Sheehan J.J., Roos Y.H. (2016). Carotenoid stability in high total solid spray dried emulsions with gum Arabic layered interface and trehalose–WPI composites as wall materials. Innov. Food Sci. Emerg. Technol..

[22] Lavelli V., Sereikaitė J. (2022). Kinetic Study of Encapsulated &beta;-Carotene Degradation in Dried Systems: A Review. Foods.

[23] Castro-Muñoz R., Barragán-Huerta B.E., Yáñez-Fernández J. (2015). Use of gelatin-maltodextrin composite as an encapsulation support for clarified juice from purple cactus pear (Opuntia stricta). LWT-Food Sci. Technol..

[24] Liu Y., Li X., Sun H., Zhang J., Cai C., Xu N., Feng J., Nan B., Wang Y., Liu J. (2023). Whey protein concentrate/pullulan gel as a novel microencapsulated wall material for astaxanthin with improving stability and bioaccessibility. Food Hydrocoll..

[25] Niu B., Shao P., Feng S., Qiu D., Sun P. (2020). Rheological aspects in fabricating pullulan-whey protein isolate emulsion suitable for electrospraying: Application in improving β-carotene stability. LWT.

[26] Drosou C., Krokida M., Biliaderis C.G. (2018). Composite pullulan-whey protein nanofibers made by electrospinning: Impact of process parameters on fiber morphology and physical properties. Food Hydrocoll.

[27] Oikonomopoulou V., Stramarkou M., Plakida A., Krokida M. (2022). Optimization of encapsulation of stevia glycosides through electrospraying and spray drying. Food Hydrocoll..

[28] Brunauer S., Emmett P.H., Teller E. (1938). Adsorption of Gases in Multimolecular Layers. J. Am. Chem. Soc..

[29] Berg C.v.d., Bruin S.C., Rockland L.B., Stewart G.F. (1981). Water activity and its estimation in food systems: Theoretical aspects. Water Activity: Influences on Food Quality.

[30] Mohammed N.K., Tan C.P., Manap Y.A., Alhelli A.M., Hussin A.S.M. (2017). Process conditions of spray drying microencapsulation of Nigella sativa oil. Powder Technol..

[31] Sablania V., Bosco S.J.D. (2018). Optimization of spray drying parameters for Murraya koenigii (Linn) leaves extract using response surface methodology. Powder Technol..

[32] Jain A., Thakur D., Ghoshal G., Prakash O., Shivhare U. (2015). Microencapsulation by Complex Coacervation Using Whey Protein Isolates and Gum Acacia: An Approach to Preserve the Functionality and Controlled Release of β-Carotene. Food Bioprocess Technol..

[33] Fan Y., Yi J., Zhang Y., Wen Z., Zhao L. (2017). Physicochemical stability and in vitro bioaccessibility of β-carotene nanoemulsions stabilized with whey protein-dextran conjugates. Food Hydrocoll..

[34] Ferraz M.C., Procopio F.R., Furtado G.d.F., Hubinger M.D. (2022). Co-encapsulation of paprika and cinnamon oleoresin by spray drying using whey protein isolate and maltodextrin as wall material: Development, characterization and storage stability. Food Res. Int..

[35] Corrêa-Filho L.C., Lourenço M.M., Moldão-Martins M., Alves V.D. (2019). Microencapsulation of β-Carotene by Spray Drying: Effect of Wall Material Concentration and Drying Inlet Temperature. Int. J. Food Sci..

[36] de Barros Fernandes R.V., Marques G.R., Borges S.V., Botrel D.A. (2014). Effect of solids content and oil load on the microencapsulation process of rosemary essential oil. Ind. Crops Prod..

[37] Zahran H.A., Catalkaya G., Yenipazar H., Capanoglu E., Şahin-Yeşilçubuk N. (2023). Determination of the Optimum Conditions for Emulsification and Encapsulation of Echium Oil by Response Surface Methodology. ACS Omega.

[38] Carmona P.A.O., Garcia L.C., Ribeiro J.A.d.A., Valadares L.F., Marçal A.d.F., de França L.F., Mendonça S. (2018). Effect of Solids Content and Spray-Drying Operating Conditions on the Carotenoids Microencapsulation from Pressed Palm Fiber Oil Extracted with Supercritical CO_2_. Food Bioprocess Technol..

[39] Fernandes R.V.d.B., Borges S.V., Botrel D.A., Silva E.K., Costa J.M.G.d., Queiroz F. (2013). Microencapsulation of Rosemary Essential Oil: Characterization of Particles. Drying Technol..

[40] Čulina P., Zorić Z., Garofulić I.E., Repajić M., Dragović-Uzelac V., Pedisić S. (2023). Optimization of the Spray-Drying Encapsulation of Sea Buckthorn Berry Oil. Foods.

[41] Tao Y., Tang Z., Huang Q., Xu X., Cheng X., Zhang G., Jing X., Li X., Liang J., Granato D. (2024). Effects of spray drying temperature on physicochemical properties of grapeseed oil microcapsules and the encapsulation efficiency of pterostilbene. LWT.

[42] Zhang L., Liao W., Wei Y., Tong Z., Wang Y., Gao Y. (2021). Fabrication, characterization and in vitro digestion of food-grade β-carotene high loaded microcapsules: A wet-milling and spray drying coupling approach. LWT.

[43] Drosou C.G., Krokida M.K., Biliaderis C.G. (2017). Encapsulation of bioactive compounds through electrospinning/electrospraying and spray drying: A comparative assessment of food-related applications. Drying Technol..

[44] González-Ortega R., Faieta M., Di Mattia C.D., Valbonetti L., Pittia P. (2020). Microencapsulation of olive leaf extract by freeze-drying: Effect of carrier composition on process efficiency and technological properties of the powders. J. Food Eng..

[45] Fioramonti S.A., Rubiolo A.C., Santiago L.G. (2017). Characterisation of freeze-dried flaxseed oil microcapsules obtained by multilayer emulsions. Powder Technol..

[46] Al-Maqtari Q.A., Mohammed J.K., Mahdi A.A., Al-Ansi W., Zhang M., Al-Adeeb A., Wei M., Phyo H.M., Yao W. (2021). Physicochemical properties, microstructure, and storage stability of Pulicaria jaubertii extract microencapsulated with different protein biopolymers and gum arabic as wall materials. Int. J. Biol. Macromol..

[47] Mohammed J.K., Mahdi A.A., Ma C., Elkhedir A.E., Al-Maqtari Q.A., Al-Ansi W., Mahmud A., Wang H. (2021). Application of argun fruit polysaccharide in microencapsulation of *Citrus aurantium* L. essential oil: Preparation, characterization, and evaluating the storage stability and antioxidant activity. J. Food Meas. Charact..

[48] Misra S., Pandey P., Panigrahi C., Mishra H.N. (2023). A comparative approach on the spray and freeze drying of probiotic and Gamma-aminobutyric acid as a single entity: Characterization and evaluation of stability in simulated gastrointestinal conditions. Food Chem. Advances.

[49] Cabuk B., Harsa S. (2015). Whey Protein-Pullulan (WP/Pullulan) Polymer Blend for Preservation of Viability of Lactobacillus acidophilus. Dry. Technol..

[50] Li C., Wang J., Shi J., Huang X., Peng Q., Xue F. (2015). Encapsulation of tomato oleoresin using soy protein isolate-gum aracia conjugates as emulsifier and coating materials. Food Hydrocoll..

[51] Teo A., Lam Y., Lee S.J., Goh K.K.T. (2021). Spray drying of whey protein stabilized nanoemulsions containing different wall materials—Maltodextrin or trehalose. LWT.

[52] Mihalcea L., Turturica M., Ghinea I., Barbu V., Enachi E., Cotarlet M., Nicoleta S. (2017). Encapsulation of carotenoids from sea buckthorn extracted by CO_2_ supercritical fluids method within whey proteins isolates matrices. Innov. Food Sci. Emerg. Technol..

[53] Sota-Uba I., Bamidele M., Moulton J., Booksh K., Lavine B.K. (2021). Authentication of edible oils using Fourier transform infrared spectroscopy and pattern recognition methods. Chemom. Intellig. Lab. Syst..

[54] Stępień A., Witczak M., Witczak T. (2020). Moisture sorption characteristics of food powders containing freeze dried avocado, maltodextrin and inulin. Int. J. Biol. Macromol..

[55] Deshwal G.K., Singh A.K., Kumar D., Sharma H. (2020). Effect of spray and freeze drying on physico-chemical, functional, moisture sorption and morphological characteristics of camel milk powder. LWT.

[56] Pavón-García L.M.A., Pérez-Alonso C., Orozco-Villafuerte J., Pimentel-González D.J., Rodríguez-Huezo M.E., Vernon-Carter E.J. (2011). Storage stability of the natural colourant from Justicia spicigera microencapsulated in protective colloids blends by spray-drying. Int. J. Food Sci. Technol..

[57] Carmo E.L.d., Teodoro R.A.R., Félix P.H.C., Fernandes R.V.d.B., Oliveira É.R.d., Veiga T.R.L.A., Borges S.V., Botrel D.A. (2018). Stability of spray-dried beetroot extract using oligosaccharides and whey proteins. Food Chem.

[58] Djendoubi Mrad N., Bonazzi C., Courtois F., Kechaou N., Boudhrioua Mihoubi N. (2013). Moisture desorption isotherms and glass transition temperatures of osmo-dehydrated apple and pear. Food Bioprod. Process..

[59] Muzaffar K., Kumar P. (2016). Moisture sorption isotherms and storage study of spray dried tamarind pulp powder. Powder Technol..

[60] García L., Cova A., Sandoval A.J., Müller A.J., Carrasquel L.M. (2012). Glass transition temperatures of cassava starch–whey protein concentrate systems at low and intermediate water content. Carbohydr. Polym..

[61] Guadarrama-Lezama A., Jaramillo-Flores M., Gutiérrez-López G., Pérez-Alonso C., Dorantes L., Alamilla-Beltrán L. (2014). Effects of Storage Temperature and Water Activity on the Degradation of Carotenoids Contained in Microencapsulated Chili Extract. Drying Technol..

[62] Sutter S.C., Buera M.P., Elizalde B.E. (2007). β-Carotene encapsulation in a mannitol matrix as affected by divalent cations and phosphate anion. Int. J. Pharm..

[63] Mahfoudhi N., Hamdi S. (2015). Kinetic Degradation and Storage Stability of β-Carotene Encapsulated by Freeze-Drying Using Almond Gum and Gum Arabic as Wall Materials. J. Food Process. Preserv..

[64] Haas K., Obernberger J., Zehetner E., Kiesslich A., Volkert M., Jaeger H. (2019). Impact of powder particle structure on the oxidation stability and color of encapsulated crystalline and emulsified carotenoids in carrot concentrate powders. J. Food Eng..

[65] Kha T.C., Nguyen M.H., Roach P.D., Stathopoulos C.E. (2015). A storage study of encapsulated gac (Momordica cochinchinensis) oil powder and its fortification into foods. Food Bioprod. Process..

[66] Spada J.C., Noreña C.P.Z., Marczak L.D.F., Tessaro I.C. (2012). Study on the stability of β-carotene microencapsulated with pinhão (Araucaria angustifolia seeds) starch. Carbohydr. Polym..

[67] Indrawati R., Sukowijoyo H., Indriatmoko, Wijayanti R.D.E., Limantara L. (2015). Encapsulation of Brown Seaweed Pigment by Freeze Drying: Characterization and its Stability during Storage. Procedia Chem..

[68] Chiu Y.T., Chiu C.P., Chien J.T., Ho G.H., Yang J., Chen B.H. (2007). Encapsulation of Lycopene Extract from Tomato Pulp Waste with Gelatin and Poly(γ-glutamic acid) as Carrier. J. Agric. Food Chem..

[69] Sarpong F., Zhou C., Bai J., Amenorfe L.P., Golly M.K., Ma H. (2019). Modeling of drying and ameliorative effects of relative humidity (RH) against β-carotene degradation and color of carrot (Daucus carota var.) slices. Food Sci. Biotechnol..

[70] Cano-Higuita D.M., Malacrida C.R., Telis V.R.N. (2015). Stability of Curcumin Microencapsulated by Spray and Freeze Drying in Binary and Ternary Matrices of Maltodextrin, Gum Arabic and Modified Starch. J. Food Process. Preserv..

[71] Barbosa M.I.M.J., Borsarelli C.D., Mercadante A.Z. (2005). Light stability of spray-dried bixin encapsulated with different edible polysaccharide preparations. Food Res. Int..

[72] Hojjati M., Razavi S.H., Rezaei K., Gilani K. (2011). Spray drying microencapsulation of natural canthaxantin using soluble soybean polysaccharide as a carrier. Food Sci. Biotechnol..

[73] Ranveer D.R., Gatade A., Kamble H., Sahoo A. (2015). Microencapsulation and Storage Stability of Lycopene Extracted from Tomato Processing Waste. Braz. Arch. Biol. Technol..

